# MyIntuitive telemetry identifies proficiency thresholds in robotic pancreaticoduodenectomy

**DOI:** 10.1007/s00464-026-12591-1

**Published:** 2026-02-05

**Authors:** Carolina González-Abós, Luis Guillermo Reyes, Claudia Lorenzo, Fabio Ausania

**Affiliations:** 1 Department of HBP and Transplant Surgery, Hospital Clínic de Barcelona, Barcelona, Spain; 2 University of Barcelona, Barcelona, Spain; 3 Gene Therapy and Cancer, IDIBAPS, Barcelona, Spain

**Keywords:** Robotic pancreaticoduodenectomy, Learning curve, Telemetry

## Abstract

**Background:**

Quantifying learning in robotic pancreaticoduodenectomy (RPD) can be challenging because case selection and conversions complicate the interpretation of full-procedure metrics. MyIntuitive intraoperative telemetry provides objective, process-level signals that may help to capture proficiency gains.

**Methods:**

We analysed 100 consecutive RPD attempts by a single surgeon (January 2022–August 2025). The primary outcome was Full-Case idle time from MyIntuitive telemetry among completed robotic cases. Key secondary telemetry outcomes included console time, active time, arm-specific activity, and instrument exchanges. As secondary corroboration on an intention-to-treat (ITT) basis, the probability of robotic completion (no conversion) was modelled across case order with risk-adjusted (RA)-CUSUM and spline logistic regression; conversions were characterised by timing and reason on video review.

**Results:**

Telemetry demonstrated marked efficiency gains across the curve. Idle time fell from 42 to 32 min (*P* = 0.001), console time from 470 to 395 min (*P* < 0.001), and active time from 428 to 365 min (*P* < 0.001) between the initial (cases 1–60) and proficiency (> 60) phases; Arm-4 active time decreased from 40 to 17 min (*P* = 0.027). The RA-CUSUM of idle time showed a proficiency inflection near cases 50–60. Supportive resection-phase telemetry showed shorter time to complete mesopancreas dissection (from 323 to 215 min, *P* = 0.002). On ITT analysis, conversion occurred in 17% overall, declining from 23.3 to 7.5% after case 60 (*P* = 0.033); no emergency conversions occurred. Perioperative outcomes included POPF 29%, postpancreatectomy haemorrhage 8%, ≥ Clavien–Dindo III 22%, reoperation 9%, 90-day readmission 9%, 90-day mortality 2%, and median length of stay 16 days.

**Conclusion:**

A telemetry-based primary endpoint (MyIntuitive idle time) sensitively tracks the RPD learning curve and, with risk-adjusted CUSUM, identifies a proficiency threshold around ~ 60 cases. Secondary ITT completion trends and conversion mapping corroborate the telemetry signal. This telemetry-first framework provides objective, phase-specific insight to guide coaching and programme maturation in complex robotic pancreatic surgery.

**Supplementary Information:**

The online version contains supplementary material available at https://doi.org/10.1007/s00464-026-12591-1.

The number of robotic pancreaticoduodenectomies (RPD) has increased worldwide in recent years; however, fewer than 4.5% of pancreaticoduodenectomies (PD) are currently performed using a minimally invasive approach [1, 2]. Morbidity after PD remains high—even in high-volume centres—and continues to be a major concern [3]. Most complications are related to pancreatic factors that cannot be modified preoperatively [4, 5], which, combined with the technical difficulty of PD, makes the adoption of RPD challenging and the objective assessment of proficiency thresholds difficult using conventional outcomes alone.

Several studies have sought to characterise technical difficulty. The PD-ROBOSCORE was developed to quantify difficulty by incorporating patient and disease characteristics, including high body mass index (BMI), borderline resectable tumours, uncinate process location, pancreatic duct size < 4 mm, American Society of Anesthesiologists (ASA) class ≥ 3, and hepatic artery originating from the superior mesenteric artery [6]. These factors have been associated with longer operative time and a higher rate of postoperative pancreatic fistula; however, some overlap with fistula risk factors (e.g. high BMI, small duct) may confound interpretation and thus blur where true learning-related proficiency is achieved [4].

The learning curve for RPD is lengthy, with 15, 62, and 84 procedures proposed to achieve feasibility, proficiency, and mastery, respectively [6]. These milestones were defined using perioperative outcomes (operative time for feasibility, risk-adjusted major complications for proficiency, and textbook outcomes for mastery). A recent International Study Group for Pancreatic Surgery (ISGPS) consensus compared RPD complexity grade with centre and surgeon experience to guide patient selection during the learning phase [7]. Obesity, venous tumour contact, previous complicated upper abdominal disease, a main pancreatic duct of ≤ 3 mm , and a common bile duct of ≤ 5 mm are considered complexity risk factors that should be avoided during the learning curve if possible [7]. Nevertheless, in high-volume pancreatic centres (> 20 cases per year) that are not very high-volume (< 100 cases per year), assembling a “low-risk” learning cohort is often impractical; to progress within a reasonable timeframe, patients with technical difficulty factors frequently need to be included. This is compounded by current epidemiology in Europe, where obesity is rising and up to 35–40% of PD candidates report prior upper abdominal surgery [3, 4].

Given the combination of a long learning curve, a technically demanding operation and additional difficulty factors, progression cannot be objectively assessed by operative time and postoperative outcomes alone, as multiple mixed variables influence these measures. Variability in instrument exchanges, console idle time, and arm interactions may indicate the surgeon’s level of experience and highlight points of hesitation or uncertainty during the operation, providing a risk-adjusted, process-based signal of skill acquisition [8]. We therefore propose using intraoperative telemetry from the MyIntuitive application during RPD as an objective tool to assess the learning curve and identify proficiency thresholds by comparing cases of similar technical difficulty.

## Materials and methods

We conducted a retrospective observational study with prospectively collected telemetry and clinical data on 100 consecutive robotic pancreaticoduodenectomy (RPD) attempts performed by a single surgeon between January 2022 and August 2025 (intention-to-treat cohort). Intraoperative process data were exported automatically from the da Vinci Xi (MyIntuitive) for every attempt. All procedures were video-recorded; videos and operative notes were used to annotate operative phases and to adjudicate the timing and reason for conversion.

The decision to proceed with robotic surgery was influenced by factors such as the need for vascular reconstruction, the severity of obesity (> 40), the presence of previous severe necrotizing pancreatitis, and the availability of the robotic system.

The inclusion criteria were as follows: any indication for pancreaticoduodenectomy, a robotic surgical approach, age over 18 years, and high difficulty. Position of the patient in the RPD learning curve was collected. Technical difficulty was defined including PD-ROBOSCORE factors and other technical difficulty factors previously defined [3, 4]: (1) high body mass index (BMI), (2) right hepatic artery (RHA) from superior mesenteric artery (SMA), (3) ≤ 3 mm pancreatic duct, (4) uncinate process tumour location, (5) previous major abdominal surgery, (6) preoperative pancreatitis with collections at computed tomography (CT) scan, (7) preoperative cholangitis requiring antibiotic treatment or repeated biliary stenting, and (8) tumour contact with portal vein and/or superior mesenteric vein (PV–SMV).

All procedures were performed with Da Vinci Xi System, as previously described [4, 5]. The robotic technique followed a standardised protocol throughout the learning curve. Any minor refinements introduced from the initial to the final cases are described in the Results section. The decision to perform an SMA-first approach was individualised and was used in the following situations: (1) suspected or confirmed PDAC, (2) prior pancreatitis, (3) uncinate process tumours, (4) suspected anomalous arterial anatomy, (5) prominent inferior pancreaticoduodenal artery, (6) hypertrophic gastroduodenal artery, and (7) suspected venous contact. In patients with PDAC, resection of the retroportal lamina was performed more extensively to ensure oncologic clearance, including lymphadenectomy along the SMA. All patients underwent preoperative cardiopulmonary exercise test and prehabilitation according to risk assessment [9].

All surgery videos were fully captured, and all data generated by the da Vinci Xi system were recorded in the MyIntuitive app. These data were collected retrospectively from my.intuitive.com. The surgical procedure was classified according to the operative date instead of the tag stored in MyIntuitive.

All the operations were performed by a single surgeon (FA) with large previous experience both in open (> 1000) and minimally invasive (> 500) hepato-pancreato-biliary operations. The surgical team was experienced and worked consistently together; all cases were performed by the same core team. Our centre is a high-volume pancreatic surgery centre with > 60 pancreatic resections per year.

Complications were defined according to Clavien–Dindo classification and Comprehensive Complication Index (CCI) [10]. Pancreatic, biliary and chyle leaks were defined according to the International Study Group of Pancreatic and Liver Surgery criteria [11–13]. Pure bile leaks were also identified based on the co-occurrence of pancreatic fistula. The postoperative period was managed as previously described [4, 5]. Update alternative fistula risk score (ua-FRS) was used to classify FRS [14].

To characterise learning without fixed time-sliced exports and while minimising completion bias, we used a telemetry-primary framework with intention-to-treat (ITT) corroboration plus a conversion descriptor. MyIntuitive telemetry was analysed within three predefined operative windows. First, Full-Case per-protocol telemetry spanned from insertion of the first robotic instrument to removal of the last instrument at the end of the procedure and served as the primary analytic framework for idle time (minutes), console time, active time, arm-specific activity and instrument exchanges. The primary outcome was MyIntuitive Full-Case idle time among completed robotic procedures, modelled across chronological case order; key secondary telemetry outcomes (per-protocol, Full-Case) were console time, active time, exchange rate, and arm-specific activity proportions (including Arm-4 active time). Second, a Landmark-R (resection phase) window was defined on the basis of video review as the interval from complete kocherisation and exposure of the pancreatic head to completion of mesopancreas dissection and division of the pancreatic neck. Because a structured 30–45 min pause precedes reconstruction in our programme, Landmark-R was used to report console and active time to the end of mesopancreas dissection and whether conversion occurred during resection and was treated as supportive, descriptive telemetry rather than as a target for risk-adjusted CUSUM. Third, for converted procedures, a Conversion window was defined from docking of the robot (or start of console time) to the time of undocking or laparotomy; for these cases, we described start-to-conversion time and the distribution of idle and active periods up to conversion, with timing and reason adjudicated on operative video. The reconstructive phase was defined on the basis of video review as the interval from pancreaticojejunal anastomosis performance to drainage placement. All procedures were video-recorded and independently annotated by two HPB surgeons (FA and CGA) for phase boundaries and exact timing of conversion events. When telemetry and video timestamps differed, video time-points were taken as reference. Robotic arm mapping was standardised across the series on the da Vinci Xi platform: Arm 2 carried the endoscope, Arms 3 and 4 were used as the two “right-hand” working arms (with Arm 4 predominantly dedicated to exposure and retraction), and Arm 1 was used for left-hand assistance. Arm-4 active time was prespecified as a learning-sensitive endpoint because it operationalises exposure/retraction workload at the console; in this mapping, Arm-4 activity concentrates during resection steps and excessive use typically co-occurs with instrument collisions, idle bursts from camera/arm re-planning, and higher exchange rates. With skill acquisition, surgeons require fewer dynamic Arm-4 adjustments, yielding shorter Arm-4 active time alongside lower idle time and exchange rates. On an ITT basis, robotic completion without conversion (success versus conversion) served as a secondary corroborative endpoint at the programme level. Clinical outcomes included operating time, estimated blood loss, conversion (timing and reason), postoperative pancreatic fistula (POPF), postpancreatectomy haemorrhage, ≥ Clavien–Dindo III complications, Comprehensive Complication Index (CCI), reoperation, length of stay, and 90-day readmission/mortality.

For the primary analysis, learning was quantified using Full-Case idle time (minutes). First, we modelled idle time as a function of case order using restricted cubic-spline regression, adjusting a priori for technical difficulty with PD-ROBOSCORE, to obtain a smooth, risk-adjusted learning curve. Second, we constructed a PD-ROBOSCORE–adjusted risk-adjusted cumulative sum (RA-CUSUM) chart of idle time. To do this, Full-Case idle time was modelled using linear regression with PD-ROBOSCORE as the only covariate; for each case, we obtained model residuals, ordered them by case number, and calculated the cumulative sum across the series. The learning-curve breakpoint and its 95% confidence interval were estimated by segmented regression fitted to the RA-CUSUM trajectory. To obtain confidence intervals, we performed nonparametric bootstrap resampling at the case level (1000 resamples), refitting the PD-ROBOSCORE-adjusted idle-time model, RA-CUSUM, and segmented regression at each draw. No propensity scores or inverse-probability weighting were used in this primary idle-time analysis. For descriptive comparisons we report a 60-case split, motivated by prior proficiency milestones [15]; the primary learning analyses were conducted continuously using spline regression and RA-CUSUM, which in our data identified an inflection around case 60.

As a secondary intention-to-treat analysis, we evaluated completion of RPD versus conversion to open surgery. First, we built an inverse-probability-weighting (IPW) model using logistic regression to estimate the probability of conversion based on baseline covariates (age, body mass index [BMI], sex, tumour location in the uncinate process, vascular involvement, replaced hepatic/superior mesenteric artery, and intraoperative pancreatic duct diameter). Stabilised weights were derived from these predicted probabilities and used to create a pseudo-population in which the distribution of covariates was balanced between converted and non-converted cases. Covariate balance was assessed using standardised mean differences (target < 0.10), the distribution of stabilised weights, and visual inspection of propensity score overlap to support the positivity assumption.

In this weighted pseudo-population, we then fitted a logistic regression model for robotic completion and, for each case, obtained the expected probability of completion. An IPW-adjusted RA-CUSUM chart was constructed by plotting, in chronological order, the cumulative sum of the difference between observed and expected completion across the series. A one-sided, improvement-oriented formulation was used, so that an upward trend indicated performance better than predicted by case-mix complexity (i.e. more completions than expected), whereas a downward trend indicated worse-than-expected performance. The breakpoint and its 95% confidence interval were estimated using segmented regression with 1000 bootstrap resamples, recalculating the stabilised inverse-probability weights and refitting the weighted logistic model and RA-CUSUM at each draw. This IPW-adjusted RA-CUSUM was used exclusively for the completion-versus-conversion endpoint.

Timing and reasons for conversions—adjudicated on operative videos and notes—were summarised descriptively and examined across case order.

Additionally, learning curves for major complications and length of stay (LOS) were evaluated using RA-CUSUM analysis. This involved estimating the expected event probabilities for each case using multivariable logistic regression models. Learning curve for LOS was evaluated using RA-CUSUM adjusted for patient age, whereas major complications were assessed using RA-CUSUM based on multivariable logistic regression models adjusted for patient age and technical difficulty expected event probabilities were estimated using multivariable logistic regression models adjusted for patient age and technical difficulty (PD-ROBOSCORE). Intraoperative blood loss was evaluated using crude CUSUM analysis, as no clear association with patient- or procedure-related covariates was observed, risk adjustment was not applied.

### Statistical analysis

Categorical data are presented as *n* (%), with between-group comparisons by *χ*^2^ or Fisher’s exact tests as appropriate. Continuous data are summarised as mean (SD) if approximately normal or median (IQR) otherwise; between-group comparisons used the *t* test or Mann–Whitney *U* test, respectively. Two-sided *P* < 0.05 was considered statistically significant, and effect sizes are reported with 95% confidence intervals. Modelling and figure generation were performed in R (restricted cubic-spline regression, risk-adjusted CUSUM, inverse-probability weighting, and diagnostic checks); descriptive statistics were verified in IBM SPSS Statistics (Version 27 for Macintosh). Missing data were minimal and analysed by a complete-case approach; no imputation was undertaken.

This research was conducted in accordance with the protocol, the principles of the latest revised version of the Declaration of Helsinki (Seoul, 2008), the standards of Good Clinical Practice as outlined by the Harmonised Tripartite Standards of the ICH for Good Clinical Practice (1996), and the guidelines for Good Epidemiological Practice (http://www.ieaweb.org/GEP07.htm). The study was evaluated and approved by the Clinical Research Ethics Committee (CEIC) of the Hospital Clinic of Barcelona (HCB/2024/0924).

## Results

All 100 consecutive RPD attempts contributed to the analysis spanning January 2022–August 2025. Median age was 69 years, median BMI was 25.7 kg/m^2^, 61% were male, and 42% had ASA ≥ III. Baseline features are shown in Table 1. All procedures used the da Vinci Xi.

**Table 1:** Baseline and intraoperative characteristics

Variable	Overall	Phase 1, 1–60	Phase 2, 61–100	*P*
Age (years), median (i.q.r)	69 (59–76)			0.747
Sex, male	61 (61.0)	40 (66.7)	21 (52.5)	0.212
BMI	26 (23–29)	25.5 (22.5–28.0)	26.8 (23–29)	0.326
ASA grade I-II	58 (58.0)	36 (60.0)	22 (55.5)	0.228
ua-FRS				0.687
Low	1 (1.0)	1(1.6)	0 (0)	
Medium	27 (28.0)	15 (25.0)	12 (30.0)	
High	72 (72.0)	44 (73.0)	28 (70.0)	
Texture, soft	75 (75.0)	44 (73.3)	31 (77.5)	0.647
Pancreatic duct diameter (mm), median (i.q.r)	2 (1–2)	2 (1–2)	2 (1–2)	0.751
Console time (min), median (i.q.r)	434 (400–479)	470 (441–532)	395 (381–412)	< 0.001
Active time (min), median (i.q.r)	397 (366–439)	428 (401–476)	365 (346–383)	< 0.001
Skin-to-skin time (min), median (i.q.r)	463 (425–508)	507 (470–565)	411 (393–426)	< 0.001
Conversion	17 (17.0)	14 (23.3)	3 (7.5)	0.033

*BMI* body mass index, *ASA* American Society of Anesthesiologists (ASA) physical status classification, *ua-FRS* update alternative fistula risk score

The operative workflow was standardised from the outset, with experience-driven refinements introduced over time. These included:

1. Trocar placement in obese patients: Port positions were shifted caudally to avoid the highest point of the pneumoperitoneum-induced abdominal dome and to improve instrument angulation.
2. SMA handling: In the initial cases, the SMA was routinely identified; subsequently, full SMA exposure (SMA-first approach) was performed selectively according to the indications detailed in the Methods.
3. Right colic vein/Henle’s trunk sequence in obese patients: After gaining experience, the right colic vein was approached after the Kocher manoeuvre to achieve complete exposure of the pancreatic head, rather than completing this step before division at Henle’s trunk.
4. Use of Cadiere forceps in Arm 3: A Cadiere forceps was adopted in Arm 3 for mobilisation of the first jejunal limb, release of the ligament of Treitz, and bowel handling during reconstruction, reducing the risk of bowel injury when using the Maryland bipolar forceps or needle driver.
5. Drain placement: Drains were increasingly placed robotically.

Reconstruction was tailored to gland characteristics. Pancreatic anastomosis techniques varied according to pancreatic texture and duct size. From approximately case 40, in patients with a friable gland, we adopted a continuous double-layer pancreaticojejunostomy using 4/0 V-Loc for the outer layer and a 5/0 Prolene duct-to-mucosa anastomosis to reduce parenchymal bleeding. Biliary anastomosis technique did not change throughout the learning curve. For the gastroenteric anastomosis, a SureForm stapler was used from around case 40; positioning the stapler in Arm 1 facilitated manoeuvrability.

Cases were stratified into an initial phase (Group A: cases 1–60) and a proficiency phase (Group B: cases > 60). Mean PD-ROBOSCORE was 9.2 in Group A versus 8.9 in Group B and a high PD-ROBOSCORE was balanced between groups (50.0% vs. 44.1%; *P* = 0.591). PD-ROBOSCORE > 12 was associated with a higher risk of conversion (10.9% vs. 27.8%; *P* = 0.032). Console time was longer in high- versus lower-risk cases (448 vs. 416 min; *P* = 0.042).

The overall conversion rate was 17% (23.3% in Group A vs. 7.5% in Group B; *P* = 0.033), with no emergency conversions. Conversions clustered during the resection phase (e.g. tunnelling at the pancreatic neck, uncinate–mesopancreas dissection) as shown in the phase-timing plot (Fig. 3). Reasons included severe adhesions for pancreatitis (*n* = 5), vascular tumour contact at the pancreatic tunnel (*n* = 5), inability to separate safely the uncinate process from SMV (*n* = 4), infiltration of a replaced right hepatic artery from the SMA (*n* = 1), SMV infiltration (*n* = 1), and Henle’s trunk infiltration (*n* = 1). In start-to-conversion analyses, median console time to conversion was 246 min with no trend across case order; blood loss in converted cases did not differ significantly from the overall cohort. Idle/active distribution up to conversion was verified on video review.

Perioperative outcomes were as follows: only one patient had > 400 mL intraoperative blood loss; POPF 29% (ISGPS; grade breakdown in Table 2); postpancreatectomy haemorrhage 8%; ≥ Clavien–Dindo III 22%; reoperation 9%; 90-day readmission 9%; 90-day mortality 2% (two patients aged 80 and 83 years with POPF complicated by sepsis). Median hospital stay was 16 days (IQR 8–28).

**Table 2:** Comparison of postoperative outcomes in both phases

Variable	Overall	Phase 1, 1–60	Phase 2, 61–100	*P*
CR-POPF (grade B/C), *n* (%)	29 (29.0)	18 (30.0)	11 (27.5)	0.575
CR-POPF (grade C), *n* (%)	2 (2.0)	1 (1.6)	1 (2.5)	0.449
DGE (grade B/C), *n* (%)	20 (20.0)	13 (21.6)	7 (17.6)	0.320
PPH (grade B/C), *n* (%)	8 (8.0)	6 (10.0)	2 (5.0)	0.167
Bile leak (grade B/C), *n* (%)	4 (4.0)	3 (5.0)	1 (2.5)	0.469
Chyle leak (grade B/C), *n* (%)	0 (0)	0 (0)	0 (0)	–
CD ≥ 3, *n* (%)	22 (22.0)	14 (23.3)	8 (20.0)	0.929
Median CCI	17.3	17.1	18.1	0.636
Reoperation	9 (9.0)	6 (10.0)	3 (7.5)	0.502
Hospital stay, days median, IQR	16 (8–28)	17 (9–28)	16 (8–25)	0.907
Readmission within 30 days *n* (%)	9 (9.0)	6 (10.0)	3 (7.5)	0.502
90-day mortality, *n* (%)	2 (2.0)	2 (3.3)	0 (0)	0.207
R0 status among PDAC, *n* (%)	33 (76.7)	18 (75.0)	15 (78.9)	0.785
Type of tumour, *n* (%)				0.861
PDAC	43 (43.0)	24 (40.0)	19 (47.5)	
Distal cholangiocarcinoma	26 (26.0)	17 (28.3)	9 (22.5)	
Ampullary cancer	14 (14.0)	8 (13.3)	6 (15.0)	
Others	17 (17.0)	11 (18.3)	6 (15.0)	

*CR-POPF* clinically relevant postoperative pancreatic fistula, *DGE* delayed gastric emptying, *PPH* postpancreatectomy haemorrhage, *R0* negative margin for PDAC, *PDAC* pancreatic ductal adenocarcinoma

**Fig. 1:**
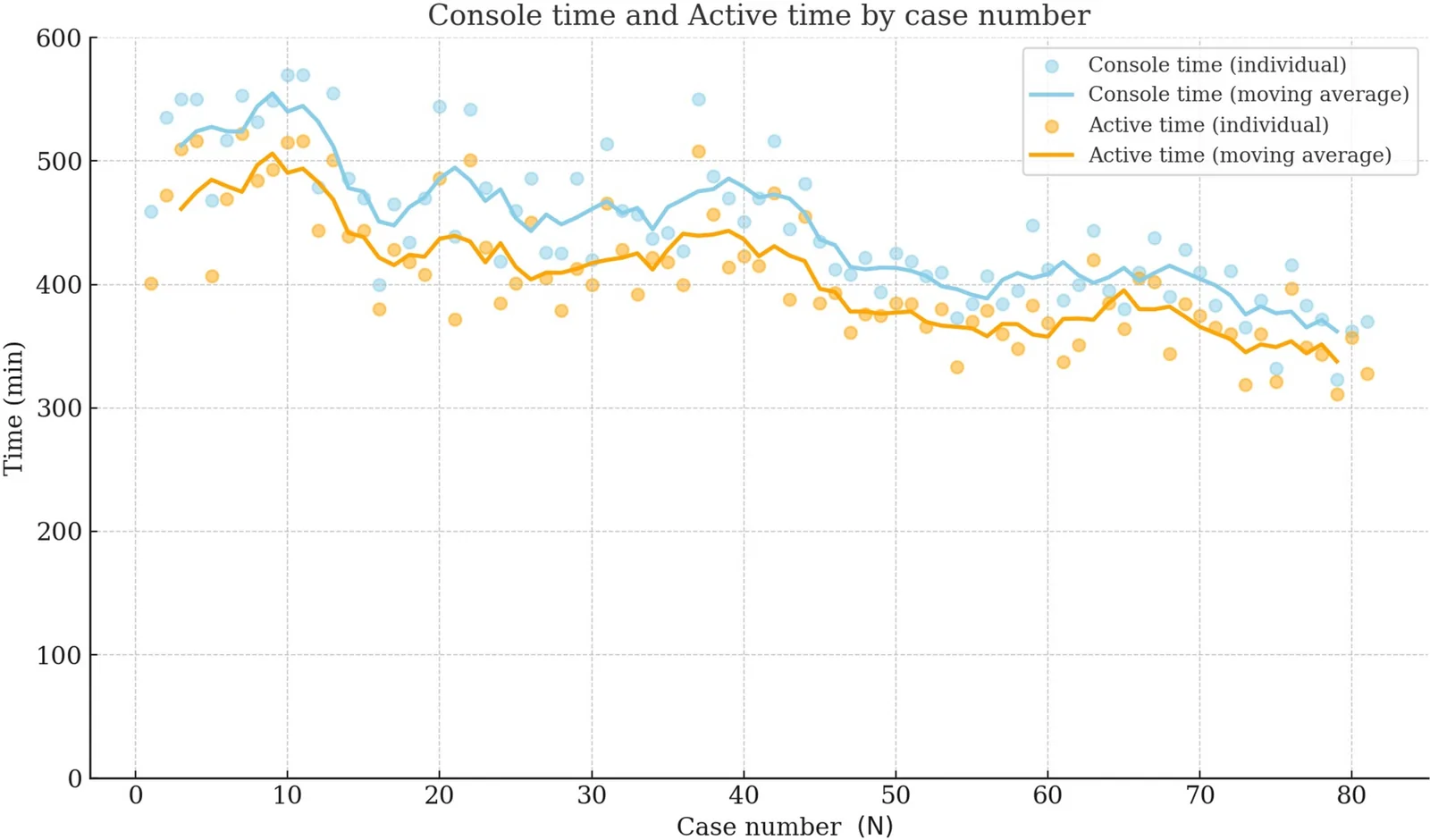
Comparison of Full-Case times of robotically completed procedures across the learning curve. Console time (blue) and active time (orange) of each case and their moving average (Color figure online)

In the per-protocol telemetry group (Full-Case), the median console time was 434 min (IQR 400–479) overall and differed between phases (470 in Group A vs. 395 in Group B; *P* < 0.001), with a clear decreasing trend across the curve (Fig. 1). Median active time was 397 min (IQR 366–439) (428 vs. 365; *P* < 0.001) (Fig. 1). The assessment of Resection-phase telemetry (Landmark-R) showed that time to complete mesopancreas dissection decreased across the curve (323 vs. 215 min; *P* = 0.002) and the proportion of total case time spent in resection increased from 32 to 37% without reaching statistical significance.

**Fig. 2:**
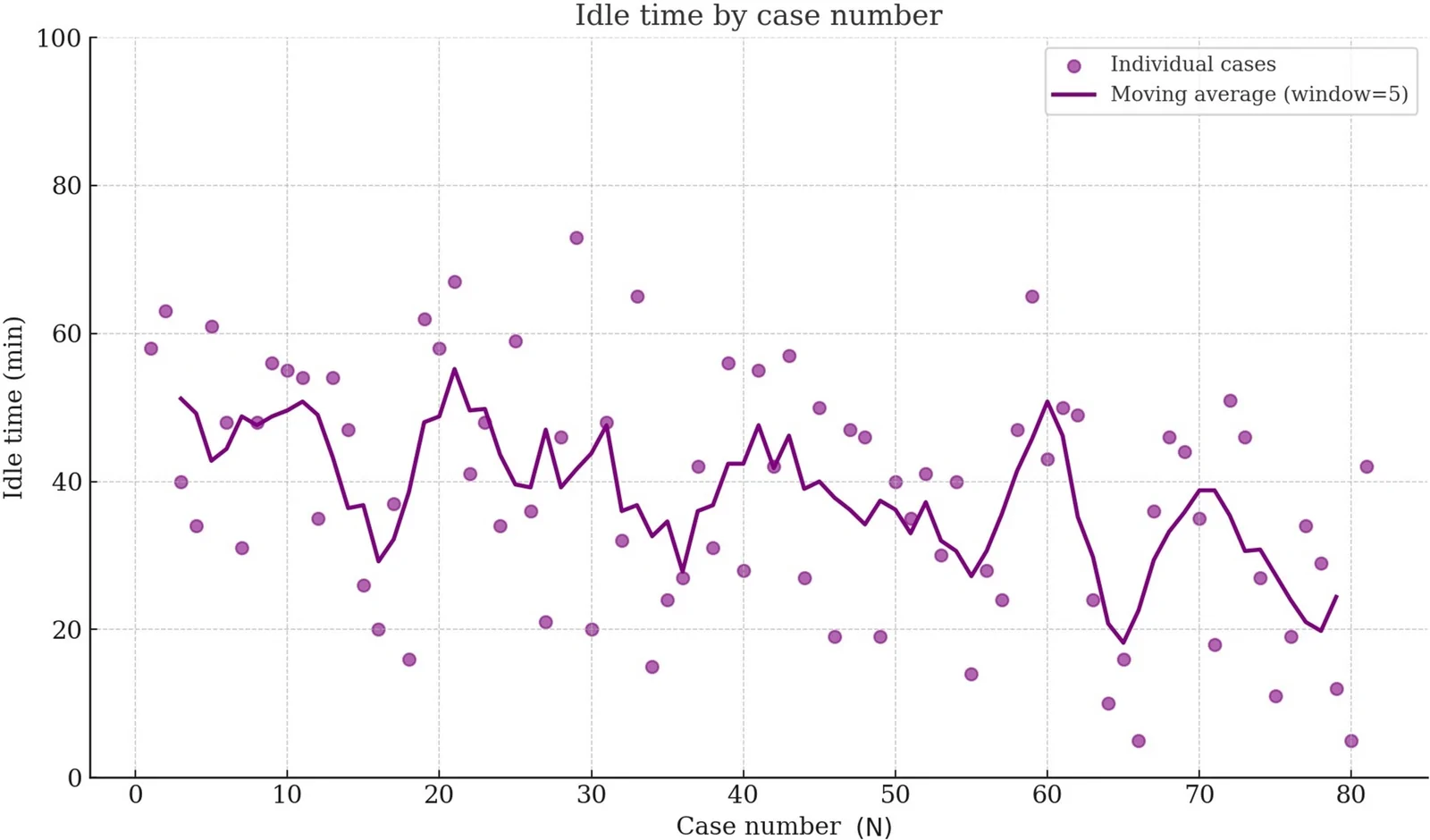
Comparison of idle time of robotically completed procedures across the learning curve

Median Full-Case idle time, the primary endpoint, was 38 min (IQR 27–47) and fell from 42 to 32 min between the initial (cases 1–60) and proficiency (> 60) phases (*P* = 0.001), with concordant reductions in console time (470 vs. 395 min; *P* < 0.001) and active time (428 vs. 365 min; *P* < 0.001) (Table 1, Figs. 1, 2). The mean number of instrument exchanges per case was 114(range 93–152), with a mean of 13 distinct instruments used (11–14). Mean Arm-4 active time (Tip-Up) was 28 min (17–44), and mean clip use per case was 19 medium (15–28) and 7 large (4–13) Hem-o-Lok® clips; overall instrument utilisation is summarised in Table 3.

**Table 3:** Comparison of active time (instruments) in both phases

Instrument metrics	Overall	Phase 1, 1–60	Phase 2, 61–100	*P* value
Instrument exchange, mean (range)	114 (93–152)	136 (80–168)	97 (57–137)	0.136
Number of instruments, mean (range)	13 (11–14)	14 (10–16)	11 (9–13)	0.076
Synchroseal, min, mean (range)	129 (121–173)	143 (118–275)	112 (98–182)	0.271
Bipolar forceps, min, mean (range)	366 (327–512)	370 (352–512)	338 (295–387)	0.845
Maryland bipolar, min, mean (range)	107 (76–125)	112 (87–148)	68 (58–91)	0.340
Cadiere, min, mean	36 (22–45)	35 (21–40)*	37 (27–46)	0.853
Tip-up, min, mean (range)	28 (17–44)	40 (21–60)	17 (9–26)	**0.027**
Scissors, min, mean (range)	14 (6–20)	21 (9–27)	9 (6–16)	0.400
Hook, min, mean (range)	12 (3–31)	18 (15–23)	8 (5–12)	0.353
Needle driver, min, mean (range)	82 (72–152)	102 (64–156)	65 (51–83)	0.095
Large clips, mean (range)	7 (4–13)	8 (7–13)	5 (4–11)	0.428
Medium clips, mean (range)	19 (15–28)	21 (18–27)	19 (15–23)	0.786
Sureform, min, mean (range)	3 (2–5)	4 (3–5)*	3 (2–4)	0.798

Bold indicates statistically significant

Only fully robotically completed cases were included for the analysis

*The use of the Cadiere forceps and sureform were introduced around case 40. Median time was calculated only in the patients where those instruments were used

**Fig. 3:**
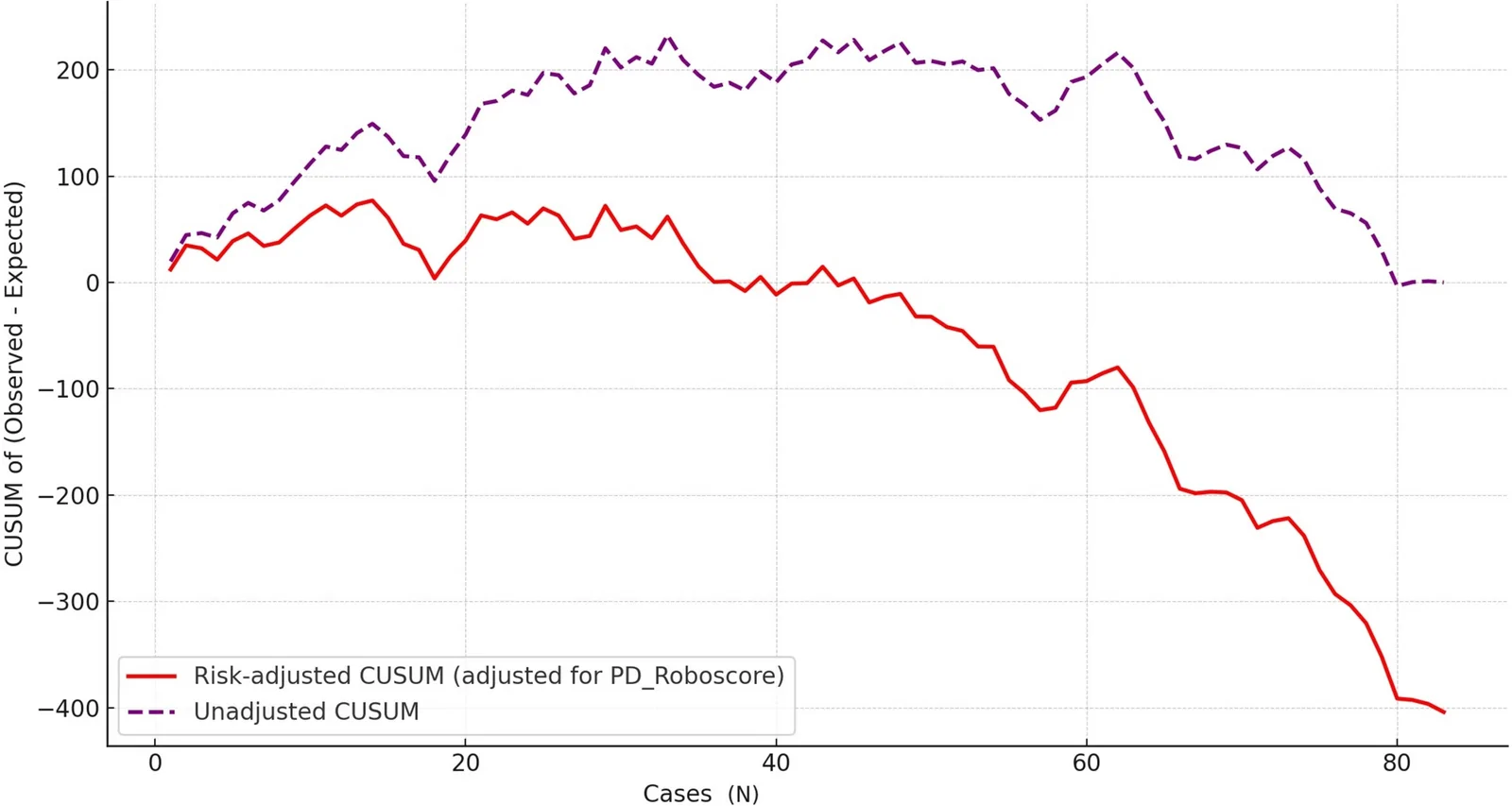
Comparison of risk-adjusted CUSUM (red) versus unadjusted CUSUM (purple) of idle time along the learning curve, adjusted to PD-ROBOSCORE (Color figure online)

On the PD-ROBOSCORE–adjusted RA-CUSUM plot of Full-Case idle time, the curve rose steeply during the first half of the series and then flattened and declined after case 60, indicating a reduction in excess idle time once proficiency had been achieved (Fig. 3). Segmented regression identified a single inflection on this RA-CUSUM trajectory in case 60, with a relatively narrow bootstrap 95% confidence band centred in the middle of the series. These include the standardised residual distributions, the leverage values, and the fitted observed plots, which are used to assess the model fit. No inverse-probability weighting or propensity score adjustment was applied, since PD-ROBOSCORE already captures procedural difficulty at the case level. We used a one-sided RA-CUSUM improvement based on standardised residuals from the adjusted idle-time model (Idle_time ~ PD-ROBOSCORE). The reference value *k* = 0.5 (corresponding to a half-standard deviation shift to detect) and the decision limit *h* = 4.3 were prespecified and calibrated by bootstrap simulation (5000 runs from the fitted model) to control the in-control false-alarm rate, targeting an average run length of approximately 500 cases (ARL 0 ≈ 500).

**Fig. 4:**
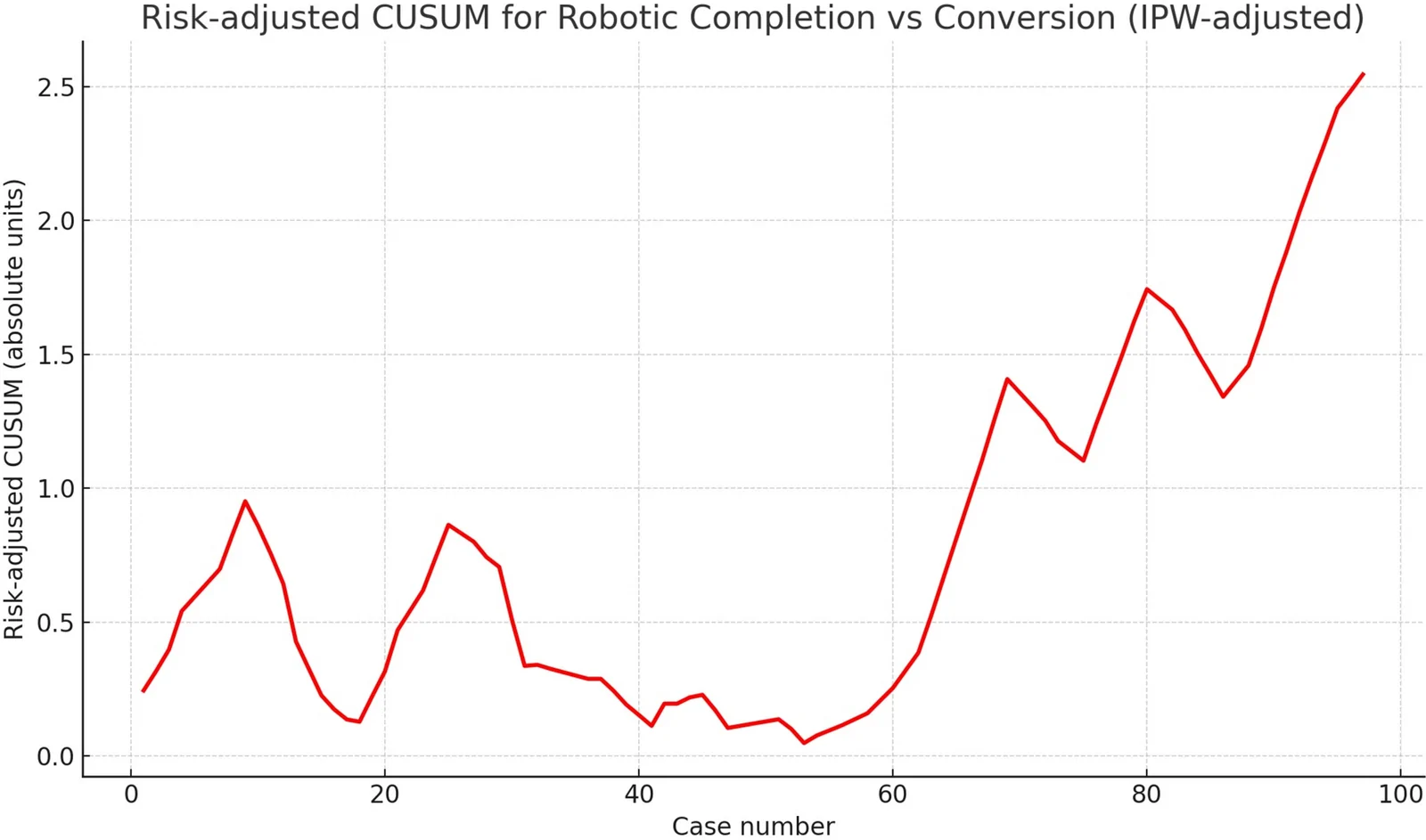
Risk-adjusted CUSUM curve for robotic completion (IPW with intraoperative variables)

In the IPW-weighted model of conversion, the variable most strongly associated with intraoperative conversion was BMI OR 1.20 (95% CI 1.01–1.43), *P* = 0.044 with higher values increasing the likelihood of conversion. Tumours located in the uncinate process showed a tendency towards higher conversion rates without reaching statistically significance (OR = 3.21 (0.76–13.49); *P* = 0.109). However, duct diameter, age, and sex did not have a significant effect (Table 4). The risk-adjusted CUSUM curve for robotic completion, which was adjusted using inverse-probability weighting (IPW) for preoperative clinical variables (age, BMI, sex, tumour location, RHA/SMA involvement, vascular involvement and duct diameter during surgery), showed an upward trend as cases were added in chronological order (Fig. 4). The cumulative sum remained relatively stable during the initial cases, followed by a sustained ascent indicating a consistent reduction in conversion rates relative to the expected performance based on case complexity. The curve’s smooth trajectory suggests a gradual and consolidated improvement in the ability to complete procedures robotically as experience increased, indicating a continuous learning effect rather than abrupt shifts in performance.

**Table 4:** IPT-weighted model of conversion

	Beta	SE	z	*P* value	CI_low	CI_high	OR	OR_CI_low	OR_CI_high
Const	− 13.0581	4.235	− 3.0834	0.002	− 21.3585	− 4.7578	0.0	0.0	0.0086
Age	0.0461	0.0341	1.3512	0.1766	− 0.0208	0.113	1.0472	0.9794	1.1197
BMI	0.1811	0.0897	2.0192	**0.0435**	0.0053	0.3569	**1.1985**	**1.0053**	**1.4289**
Sex	0.8222	0.7139	1.1518	0.2494	− 0.5769	2.2214	2.2756	0.5616	9.2202
Uncinate tumour	1.1691	0.7312	1.5989	0.1098	− 0.264	2.6023	3.2192	0.768	13.4944
Wirsung diameter	0.1944	0.1253	1.5512	0.1209	− 0.0512	0.4399	1.2145	0.9501	1.5526

Bold indicates independently associated to conversion

*CI* confidence interval, *OR* odds ratio, *BMI* body mass index

Further analysis of the learning curve showed that LOS stabilised at case 28. An RA-CUSUM analysis of LOS, adjusted for patient age, revealed a clear temporal trend. During the initial phase, the RA-CUSUM curve showed a pronounced downward trend, indicating that the observed LOS was consistently shorter than expected for the patient’s age group (see, Fig. S1A). This initial improvement continued until approximately case 28, after which a change in slope was observed. Beyond this point, the RA-CUSUM curve ceased to decrease, instead exhibiting a mild upward drift with increased variability. This reflects LOS values that, on average, are slightly longer than expected. Overall, these findings suggest an initial improvement in postoperative LOS, followed by stabilisation without further sustained reduction. At last, RA-CUSUM analysis of major complications, adjusted for age and BMI, showed an initial phase with fewer complications than expected, followed by a transition around 30 cases and late stabilisation thereafter (see, Fig. S1B). In contrast, crude CUSUM analysis of intraoperative blood loss did not demonstrate a clear temporal trend, with the curve remaining relatively stable throughout the series, indicating consistent blood loss over time (see, Fig. S1C).

## Discussion

This single-centre series is, to our knowledge, the first comprehensive evaluation of automatically captured MyIntuitive intraoperative telemetry applied to robotic pancreaticoduodenectomy (RPD). The central finding is that the telemetry-based primary endpoint—Full-Case idle time (minutes)—tracks the learning curve and shows a risk-adjusted inflection around cases 60 on a continuous RA-CUSUM of model residuals. Efficiency gains observed in per-protocol Full-Case analyses (declines in console and active times) and the secondary ITT trend towards increasing robotic completion without conversion provide convergent validity while limiting completion bias.

To date, learning curves in pancreatic surgery have largely been assessed using intraoperative and postoperative outcomes, often without capturing the full procedure as an integrated workflow (including technique evolution, instrumentation, and operative strategy) [16]. Telemetry remains underused in complex surgery [8]. Unlike downstream clinical outcomes—which are infrequent, delayed, and sensitive to patient heterogeneity—process-level metrics (idle time, arm-specific activity, exchange rates) are available in every case and respond rapidly to incremental improvements. In our cohort, Full-Case idle time decreased across case order, and a risk-adjusted CUSUM built from standardised residuals identified a reproducible performance breakpoint, consistent with a transition from early inefficiency to more economical performance. Complementary reductions in console and active times among completed procedures corroborate the primary signal.

We observed a significant reduction in Arm-4 active time (40–17 min), which is consistent with a more efficient exposure/retraction process once the camera choreography, port geometry, and tissue handling have become routine. At the resection-phase landmark (Landmark-R), the time taken for mesopancreas dissection shortened materially (323–215 min). Conversely, the time spent in the reconstructive phase decreased more drastically, most likely because this part of the procedure is easier to standardise and carries less haemorrhagic risk.

Using telemetry to assess technical improvement on the learning curve seems to be a better tool than analysing perioperative surrogates directly. In our study, the stabilisation of the major complication and LOS learning curves occurs around case 30, and there is no difference in blood loss during the whole learning curve. However, according to previous literature, overcoming the learning curve should occur around case 64 [6]. We believe that postoperative outcomes are closely linked to the team’s prior experience in pancreatic surgery, including the appropriate indication of surgery, the application of mitigation strategies, and the early postoperative management of patients. Our postoperative patient complications were comparable with those published in randomised controlled trials (RCTs) demonstrating RPD safety after completion of the learning curve, with fewer than 25% of major complications and 2% mortality in the whole cohort [17, 18]. When focusing on pancreatic fistula, we reported a CR-POPF rate of 29%, but there is high variability between the published studies, probably due to differences in postoperative patient management and in the definition of grade B POPF when implying modification of patient management [19]. Despite this, in our cohort, the rate of pancreatic fistula does not result in an increase in major complications. However, the LOS of our patients is higher than in some previous publications, but the readmission rate is lower [18]. Anyway, there is a difference in LOS between the different RCTs, but we hypothesise that LOS also depends on the conservatism of patient management at the different centres [20].

Additionally, including more complex cases during the intermediate phase of the learning curve can affect postoperative outcomes and create a misconception about the time it takes to acquire proficiency in the surgical technique. However, our case-selection strategy admitted substantial technical complexity from the outset—excluding only extreme obesity (BMI > 40), anticipated vascular reconstruction, or sequelae of severe necrotising pancreatitis. This pragmatic approach likely compressed calendar time to proficiency while maintaining safety: conversion fell from 23.3 to 7.5 with no emergency conversions, and perioperative outcomes were acceptable (90-day mortality 2%). The curve’s smooth trajectory of the IPW RA-CUSUM for conversion suggests a gradual and consolidated improvement in the ability to complete procedures robotically as experience increased, indicating a continuous learning effect rather than abrupt shifts in performance. PD-ROBOSCORE risk was balanced between phases; high-risk cases took longer console time (448 vs. 416 min) yet were not associated with conversion, and blood loss in converted cases was comparable to the series. While consensus statements advise deferring the most complex pathology early, strict adherence can be unrealistic in centres performing < 100 cases/year. Our data suggest that—with safeguards (phase-aware monitoring and early conversion)—measured inclusion of technically demanding cases need not compromise patient safety. As expected, conversions clustered during resection/vascular steps (e.g. pancreatic-neck tunnelling, uncinate–mesopancreas dissection); early in the programme, we deliberately maintained a low threshold for early, controlled conversion to avoid emergencies—accounting for the higher initial conversion rate and the absence of emergency conversions [21]. Although PD-ROBOSCORE > 12 was associated with higher crude conversion rates (10.9% vs. 27.8%; *P* = 0.032), this signal attenuated in the IPW-weighted model, where only BMI remained significantly associated with conversion. This is likely explained by collinearity between PD-ROBOSCORE and specific anatomical covariates included in the weighting model (such as uncinate tumours, vascular involvement, and duct diameter), together with the limited number of conversion events, which reduces the effective events-per-variable and inflates variance. In this context, the weighted analysis should be interpreted as having adjusted away much of the difficulty signal encoded in PD-ROBOSCORE rather than demonstrating that procedural complexity is unimportant. We therefore view higher BMI as a robust risk factor for conversion in this series, while PD-ROBOSCORE and its components remain clinically relevant markers of case complexity even if they do not retain statistical significance in a saturated weighted model.

A telemetry-first framework turns routine data into actionable coaching targets. In practice, programmes can set idle-time benchmarks and an Arm-4 “budget” for the resection phase, incorporate checklists for exposure/retraction choreography, and review phase timelines when RA-CUSUM inflects. Because Arm-4 behaviour is controllable (instrument choice, port layout, assistant tasks), it lends itself to straightforward interventions (e.g. stabilising retraction with gravity/bedside aids; reducing dynamic repositioning that precipitates idle bursts and exchanges). Dashboards that display idle time, Arm-specific activity, and exchange rate per active hour can provide near-real-time feedback and shorten the path to proficiency.

This study has limitations. The design is retrospective, albeit with prospectively collected telemetry and clinical data; therefore, selection bias and residual confounding remain possible despite multivariable adjustment and IPW. Findings reflect the performance of a single surgeon at a single centre, which may limit generalisability; a cohort of 100 attempts, while substantial for RPD, still yields wide intervals for uncommon events. We relied on vendor-generated MyIntuitive telemetry, which can evolve across software versions and is not yet standardised across platforms. Critically, the current application does not support time-sliced exports at user-defined landmarks (e.g. at 180 min or precisely at the end of resection), precluding quantification of instrument use or arm activity at specific landmarks; we therefore used Full-Case per-protocol telemetry and video-verified phase markers (Landmark-R) as supportive timing. Console telemetry cannot capture cognitive load, team dynamics, assistant manoeuvres, bedside ergonomics, port–bed interactions, or subtle anatomic variation. RA-CUSUM performance depends on the choice of reference/decision parameters and residual standardisation; we mitigated this with prespecification and empirical checks, but some uncertainty remains. We acknowledge that the IPW logistic model for the completion-versus-conversion endpoint was fitted on a limited number of conversion events, with covariates that partially overlap with the PD-ROBOSCORE construct; as a result, events-per-variable are low and collinearity cannot be excluded, so the IPW RA-CUSUM for completion should be interpreted as an exploratory, case-mix-adjusted corroboration of the primary idle-time analysis rather than a definitive inferential model. Finally, although ITT completion trends and weighting help limit completion bias, they do not eliminate it; team learning and institutional process changes over calendar time may also contribute to the observed trends, and the phase demarcation anchored to a routine prereconstruction pause may not be universally applicable.

The next step is multicentre validation to harmonise definitions, confirm proficiency thresholds, and establish reporting standards. Prospective, pragmatic studies should test telemetry-guided coaching—for example, setting idle time and Arm-4 targets during resection, using phase timelines and RA-CUSUM alerts—and evaluate whether adding early predictive signals (e.g. rolling idle time from recent cases) reduces conversion or operative time. Coupling telemetry with automated video phase detection, and measuring cognitive load, team dynamics, and assistant ergonomics, will enrich interpretation. Finally, programmes should address data governance and cost-effectiveness so dashboards that provide timely, objective feedback can be sustained in routine practice.

In conclusion, MyIntuitive telemetry—using Full-Case idle time (min) as the primary endpoint—captured the RPD learning curve and pinpointed a ~ 50–60 case proficiency inflection. Concordant drops in console/active time, reduced Arm-4 demand, and shorter resection phases (with ITT completion corroboration and no emergency conversions) support embedding standardised telemetry and RA-CUSUM into coaching to accelerate safe programme maturation.

## Supplementary Information

Below is the link to the electronic supplementary material.

Supplementary file1 (PNG 246 KB)—Figure S1. Learning curve analysis of intraoperative blood loss, major complications, and length of stay. (A) Risk-adjusted cumulative sum (RA-CUSUM) analysis of length of stay (LOS) adjusted for patient age, showing an initial phase with shorter-than-expected LOS followed by stabilisation. (B) RA-CUSUM analysis of major complications (Clavien–Dindo grade ≥ III) adjusted for patient age and body mass index, demonstrating an initial phase with fewer complications than expected, followed by a transition phase and late stabilisation. (C) Crude cumulative sum (CUSUM) analysis of intraoperative blood loss, showing no clear temporal trend and overall stability throughout the series. In all panels, the x-axis represents consecutive cases ordered by operative date, and changes in slope reflect shifts in performance relative to expected values.

## Data Availability

The authors confirm that the data supporting the findings of this study are available within the article and its supplementary materials.
