# Supplementary figures and images for: MyIntuitive telemetry identifies proficiency thresholds in robotic pancreaticoduodenectomy
Source: Surg Endosc. 2026 Feb 5;40(4):3284–95. doi: 10.1007/s00464-026-12591-1 (PMC13053344; doi:10.1007/s00464-026-12591-1)

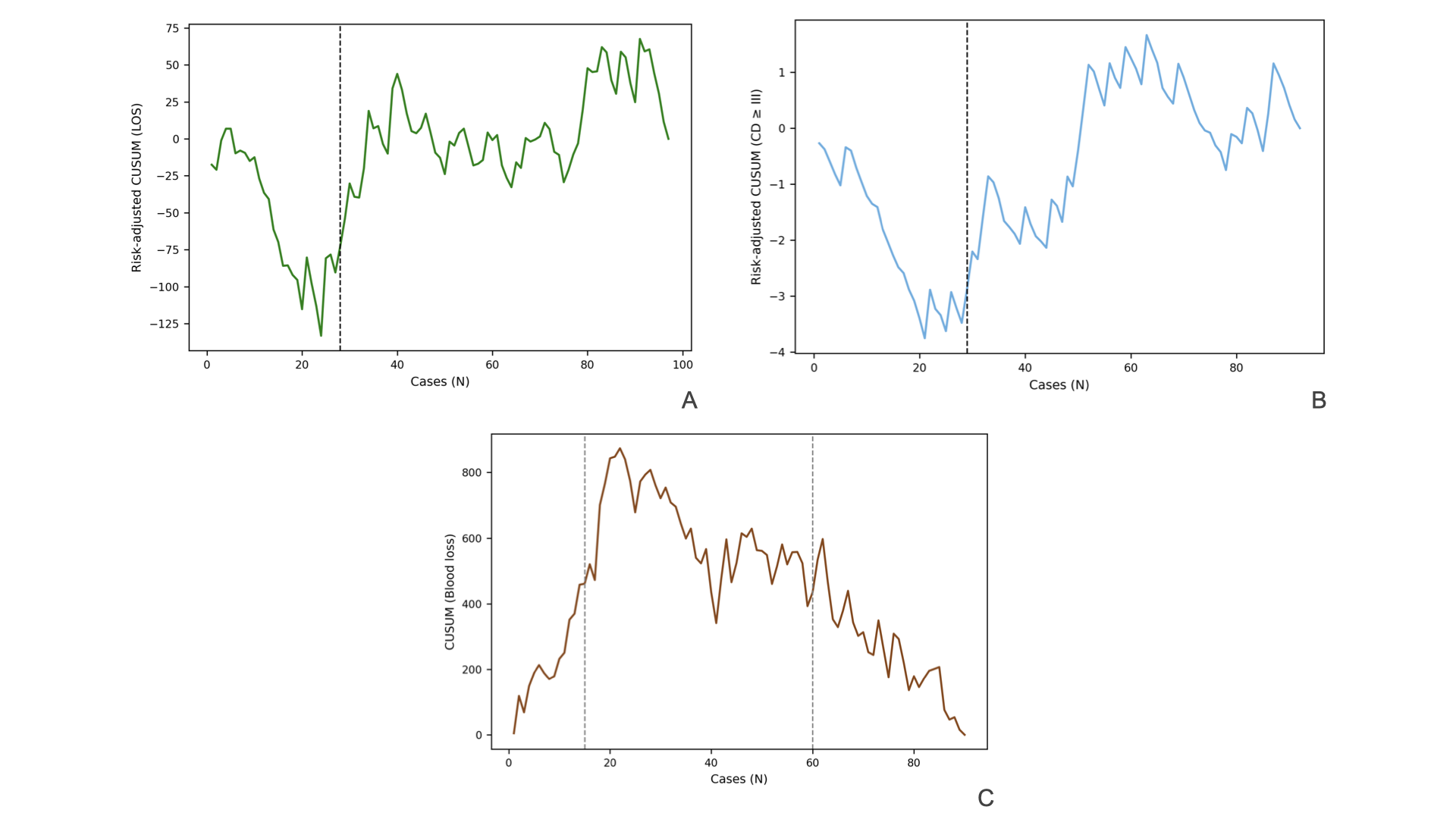

Supplement: Supplementary file 1 — Supplementary file1 (PNG 246 KB)—Figure S1. Learning curve analysis of intraoperative blood loss, major complications, and length of stay. (A) Risk-adjusted cumulative sum (RA-CUSUM) analysis of length of stay (LOS) adjusted for patient age, showing an initial phase with shorter-than-expected LOS followed by stabilisation. (B) RA-CUSUM analysis of major complications (Clavien–Dindo grade ≥ III) adjusted for patient age and body mass index, demonstrating an initial phase with fewer complications than expected, followed by a transition phase and late stabilisation. (C) Crude cumulative sum (CUSUM) analysis of intraoperative blood loss, showing no clear temporal trend and overall stability throughout the series. In all panels, the x-axis represents consecutive cases ordered by operative date, and changes in slope reflect shifts in performance relative to expected values. [file 464_2026_12591_MOESM1_ESM.png]
